# Characteristics of Differently Located Colorectal Cancers Support Proximal and Distal Classification: A Population-Based Study of 57,847 Patients

**DOI:** 10.1371/journal.pone.0167540

**Published:** 2016-12-09

**Authors:** Jiao Yang, Xiang lin Du, Shu ting Li, Bi yuan Wang, Yin ying Wu, Zhe ling Chen, Meng Lv, Yan wei Shen, Xin Wang, Dan feng Dong, Dan Li, Fan Wang, En xiao Li, Min Yi, Jin Yang

**Affiliations:** 1 Department of Medical Oncology, The First Affiliated Hospital of Xi’an Jiaotong University, Xi’an city, Shaanxi Province, China; 2 Department of Epidemiology, Human Genetics, and Environmental Sciences, The University of Texas School of Public Health, Houston, TX, United States of America; 3 Department of Breast Surgical Oncology, The University of Texas MD Anderson Cancer Center, Houston, TX, United States of America; University of Michigan, UNITED STATES

## Abstract

**Background:**

It has been suggested that colorectal cancer be regarded as several subgroups defined according to tumor location rather than as a single entity. The current study aimed to identify the most useful method for grouping colorectal cancer by tumor location according to both baseline and survival characteristics.

**Methods:**

Cases of pathologically confirmed colorectal adenocarcinoma diagnosed from 2000 to 2012 were identified from the Surveillance, Epidemiology, and End Results database and categorized into three groups: right colon cancer (RCC), left colon cancer (LCC), and rectal cancer (ReC). Adjusted hazard ratios for known predictors of disease-specific survival (DSS) in colorectal cancer were obtained using a Cox proportional hazards regression model.

**Results:**

The study included 57847 patients: 43.5% with RCC, 37.7% with LCC, and 18.8% with ReC. Compared with LCC and ReC, RCC was more likely to affect old patients and women, and to be at advanced stage, poorly differentiated or un-differentiated, and mucinous. Patients with LCC or ReC had better DSS than those with RCC in subgroups including stage III or IV disease, age ≤70 years and non-mucinous adenocarcinoma. Conversely, patients with LCC or ReC had worse DSS than those with RCC in subgroups including age ˃70 years and mucinous adenocarcinoma.

**Conclusions:**

RCC differed from both LCC and ReC in several clinicopathologic characteristics and in DSS. It seems reasonable to group colorectal cancer into right-sided (i.e., proximal) and left-sided (i.e., distal) ones.

## Introduction

Colorectal cancer (CRC) is the third most common cancer worldwide and the second most common cause of cancer-related death in western countries [1]. In clinical practice, different manifestations have been observed in CRC patients with tumors originating from different sub-sites of the colorectum. Tumors arising from the proximal colon tend to present with subtle signs and systemic symptoms such as microcytic anemia and weight loss, whereas tumors arising from the distal colorectum tend to present with local symptoms including alterations in bowel habits and rectal bleeding[2–4]. A higher proportion of the serrated lesions have been detected by endoscopy in the right colon than in the left colon and rectum [5]. Some risk factors for CRC are also site-specific. A high-fiber diet was related to decreased colon cancer risk[6],while processed red meat was associated with an increased risk for left colon cancer (LCC)[7]. Additionally, patients with ReC and LCC had significantly higher overall metastasis or recurrence rates after curative surgery when compared to RCC patients[8, 9]. In short, kinds of evidence suggests that CRC be regarded as several subgroups according to tumor location rather than as a single entity[10].

Practically, CRC has always been divided into colon cancer and rectal cancer (ReC), each of which has distinct diagnostic and therapeutic guidelines. Some recent studies found differences in clinicopathologic and prognostic features between patients with right colon cancer (RCC) and those with LCC[11–13]. Therefore, a three-part division into RCC, LCC, and ReC was proposed. Additionally, it also seems reasonable to divide CRC into right-sided (i.e., proximal) tumors and left-sided (i.e., distal) tumors, because of the differences in embryonic origin, blood supply, innervation, lymphatic drainage, and lumen environment. However, it is not well known which one was the most reasonable and useful division method in clinical practice among the three ones listed above. Therefore, the current study aimed to determine how best to group CRCs by tumor location according to both baseline and survival characteristics.

## Patients and Methods

Data were obtained from all 18 U.S. cancer registries participating in the Surveillance, Epidemiology, and End Results (SEER) Program using the SEER*Stat software program (version 8.2.1; http://seer.cancer.gov/seerstat; accessed January 2, 2016) under a data use agreement. SEER was used to retrospectively identify patients whose primary tumor sites were coded as C18.0, C18.2, C18.3, C18.4, C18.5, C18.6, C18.7, C19.9, or C20.9 (indicating CRC) and whose cancers were diagnosed from 2000 to 2012. Patients with the histologic type code ICD-O-3, indicating adenocarcinoma (code 8140–8141, 8143–8144, 8210, 8220–8221, 8255, 8260–8263, 8310, 8323, 8480–8481, 8490, 8503, 8570, and 8574), were included.

Cases were categorized by primary tumor site into three groups: RCC (C18.0: cecum, C18.2: ascending colon, C18.3: hepatic flexure of colon, or C18.4: transverse colon), LCC (C18.5: splenic flexure of colon, C18.6: descending colon, C18.7: sigmoid colon, or C19.9: rectosigmoid junction), and ReC(C20.9: rectum). Patient, tumor, and treatment characteristics known or potential prognostic value in CRC were evaluated and compared among the three groups. The primary endpoint of this study was disease-specific survival (DSS), and time to DSS was calculated as the number of years between the date of diagnosis and the date of CRC-related death, the date the patient was last known to be alive, or December 31, 2012, which ever occurred first. Patients not experiencing DSS were censored at last follow-up. DSS curves were calculated using the Kaplan-Meier method. Multivariate Cox proportional hazards models were used to determine the influence of the collected patient, tumor, and treatment factors on DSS. Kruskal-Wallis equality-of-populations rank test was used to compare baseline characteristics between tumor location groups. Stata/SE version 12.1 statistical software (Stata Corp LP, College Station, TX) was used for statistical analyses. All tests were two-tailed, and statistical significance was defined as P < 0.05. In order to make it more persuasive, we made similar analyses after proportionating the three groups by random sampling 50% from RCC and LCC, and the results were provided in supplemental tables.

## Results

### Patient and tumor characteristics

A total of 57847 patients were included in this study: 25181 patients (43.5%) with RCC, 21791 patients (37.7%) with LCC, and 10875 patients (18.8%) with ReC. The median age at diagnosis was 72 years (range 17–108 years). The median follow-up time was 3.2 years (mean 4.2 years, range 0.01–12.9 years). Mucinous histology (ICD-O-3 as 8480, 8481, and 8490) was identified in 10.6% of patients. Most patients (61.6%) had grade II tumors.

**Fig 1:**
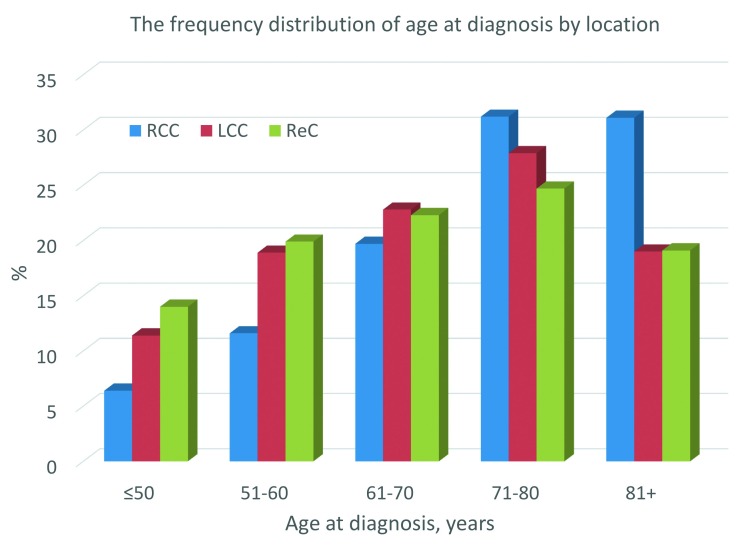
The frequency distribution of age at diagnosis by tumor location. Right colon cancer (RCC), left colon cancer (LCC), rectal cancer (ReC).

There were significant differences in baseline characteristics between tumor location groups (Table 1 and S1 Table). Compared with LCC and ReC, RCC was significantly more likely to affect older patients (median age 75 years) and women (55.4%), to be advanced stage (stage II and above; 72.1%), and to have mucinous histology (14.9%). Fig 1 showed the frequency distribution of age at diagnosis among the three location groups (Fig 1). Primary surgery for cancer was performed most frequently for RCC patients (91.2%) and least frequently in ReC patients (78.3%) (P<0.0001 for each).

**Table 1:** Baseline characteristics by primary tumor location.

Characteristic	RCC, no. (%)(n = 25181)	LCC, no. (%)(n = 21791)	ReC, no. (%) (n = 10875)	P
Age at diagnosis, years				<0.0001^
Median (range)	75 (17–108)	69 (17–102)	68 (20–107)	
≤50	1617 (6.4)	2483 (11.4)	1525 (14.0)	
51–60	2929 (11.6)	4130 (18.9)	2161 (19.9)	
61–70	4951 (19.7)	4965 (22.8)	2427 (22.3)	
71–80	7845 (31.2)	6073 (27.9)	2684 (24.7)	
81+	7389 (31.1)	4140 (19.0)	2078 (19.1)	
Year of diagnosis				<0.0001
2000–2004	12309 (48.9)	11254 (51.7)	5215 (48.0)	
2005–2008	6902 (27.4)	5715 (26.2)	3021 (27.8)	
2009–2012	5970 (23.7)	4822 (22.1)	2639 (24.2)	
Ethnicity				<0.0001
Non-Hispanic white	18798 (74.7)	15435 (70.8)	8029 (73.8)	
Black	3323 (13.2)	2491 (11.4)	1009 (9.3)	
Hispanic white	1311 (5.2)	1301 (6.0)	680 (6.3)	
Asian	1609 (6.4)	2358 (10.8)	1075 (9.9)	
Others	140 (0.5)	206 (1.0)	82 (0.8)	
Sex				<0.0001
Female	13951 (55.4)	10286 (47.2)	4510 (41.5)	
Male	11230 (44.6)	11505 (52.8)	6365 (58.5)	
Stage				<0.0001*
0	657 (2.6)	948 (4.4)	469 (4.3)	
I	5371 (21.3)	5804 (26.6)	3201 (29.4)	
II	7660 (30.5)	5029 (23.1)	2120 (19.5)	
III	6401 (25.4)	5266 (24.2)	2512 (23.1)	
IV	4082 (16.2)	3650 (16.7)	1596 (14.7)	
Unknown	1010 (4.0)	1094 (5.0)	977 (9.0)	
Tumor grade				<0.0001*
I	2212 (8.8)	2302 (10.6)	902 (8.3)	
II	15015 (59.6)	14011 (64.3)	6595 (60.6)	
III	5262 (20.9)	2623 (12.0)	1384 (12.7)	
Undifferentiated	406 (1.6)	151 (0.7)	85 (0.8)	
Unknown	2286 (9.1)	2704 (12.4)	1909 (17.6)	
Mucinous histology				<0.0001
No	21423 (85.1)	20196 (92.7)	10094 (92.8)	
Yes	3758 (14.9)	1595 (7.3)	781 (7.2)	
Primary surgery				<0.0001*
Not performed	2183 (8.7)	2065 (9.5)	2244 (20.6)	
Performed	22958 (91.2)	19679 (90.3)	8510 (78.3)	
Unknown	40 (0.1)	47 (0.2)	121 (1.1)	

Abbreviations: RCC, right colon cancer; LCC, left colon cancer; ReC, rectal cancer.

* Exclude unknown category

^ Kruskal-Wallis equality-of-populations rank test

### Survival outcomes

**Fig 2:**
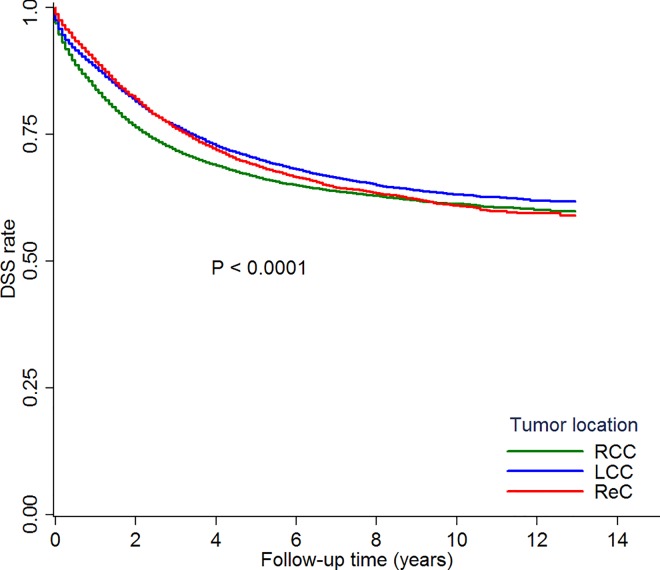
Comparison of disease-specific survival by tumor locations. Kaplan–Meier curves showing the comparisons of disease-specific survival among right colon cancer (RCC), left colon cancer (LCC) and rectal cancer (ReC), with significant difference (P<0.0001).

Univariate analysis indicated differences in outcomes between the tumor location groups (P<0.001), with 5-year DSS rates of 66.6% in the RCC group, 70.2% in the LCC group, and 68.8% in the ReC group (Fig 2). Multivariate analysis indicated that RCC location, mucinous histology, advanced stage, higher tumor grade, and black race were associated with worse DSS (S2 Table).

After adjustment for covariates, the DSSs for all stages combined were similar between the LCC and ReC groups (P = 0.99), and both the LCC (hazard ratio = 0.87, P<0.0001) and the ReC (hazard ratio = 0.94, P = 0.02) groups had significantly better DSSs than the RCC group (Table 2 and S3 Table).

**Table 2:** Cox proportional hazards analysis of influence on disease-specific survival by stage, age at diagnosis, and histologic type.

	LCC vs. RCC		ReC vs. RCC		ReC vs. LCC	
	HR (95%CI)	P	HR (95%CI)	P	HR (95%CI)	P
Overall	0.87 (0.84–0.90)	<0.0001	0.94 (0.90–0.99)	0.02	1.0 (0.95–1.05)	0.99
By stage						
I	0.81 (0.69–0.93)	0.004	1.30 (1.12–1.52)	0.001	1.61 (1.38–1.89)	<0.0001
II	1.29 (1.18–1.40)	<0.0001	1.44 (1.28–1.62)	<0.0001	1.14 (1.01–1.28)	0.04
III	0.81 (0.76–0.87)	<0.0001	0.86 (0.79–0.94)	0.001	1.08 (0.99–1.18)	0.1
IV	0.77 (0.72–0.81)	<0.0001	0.75 (0.70–0.82)	<0.0001	0.99 (0.91–1.07)	0.7
By histologic type						
Mucinous	1.28 (1.15–1.43)	<0.0001	1.20 (1.04–1.39)	0.01	0.96 (0.82–1.13)	0.6
Non-mucinous	0.94 (0.90–0.97)	0.001	0.93 (0.89–0.98)	<0.0001	1.0 (0.95–1.05)	0.9
By age at diagnosis (years)						
≤50	0.93 (0.81–1.05)	0.2	0.95 (0.81–1.10)	0.5	1.03 (0.89–1.18)	0.7
51–60	0.85 (0.77–0.94)	0.002	0.80 (0.72–0.91)	0.001	0.95 (0.84–1.07)	0.4
61–70	0.97 (0.89–1.06)	0.5	0.96 (0.86–1.07)	0.4	0.99 (0.89–1.11)	0.9
71–80	1.08 (1.01–1.15)	0.03	1.10 (1.01–1.20)	0.04	1.03 (0.94–1.14)	0.5
81+	1.18 (1.10–1.27)	<0.0001	1.17 (1.07–1.29)	0.001	1.0 (0.90–1.10)	0.96

The multivariate analysis was adjusted for race, tumor grade, and sex.

Abbreviations: RCC, right colon cancer; LCC, left colon cancer; ReC, rectal cancer; HR, hazard ratio; CI, confidence interval.

**Fig 3:**
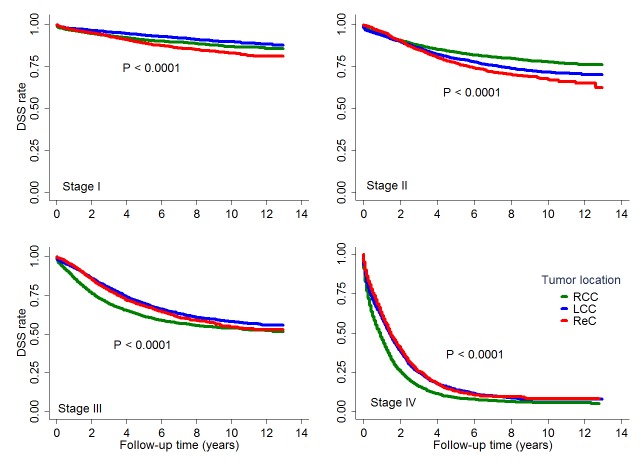
Comparison of disease-specific survival among differently located colorectal cancers stratified by stage. Kaplan–Meier curves showing the comparisons of disease-specific survival among right colon cancer (RCC), left colon cancer (LCC) and rectal cancer (ReC) within each stage, all with significant differences (all P<0.0001).

**Fig 4:**
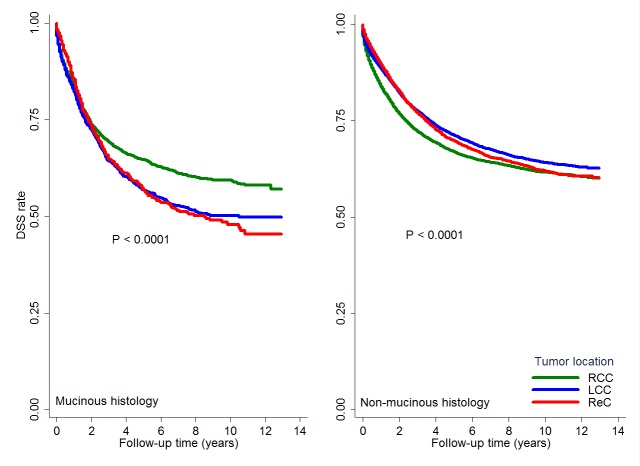
Comparison of disease-specific survival among differently located colorectal cancers stratified by histology type. Kaplan–Meier curves showing the comparisons of disease-specific survival among right colon cancer (RCC), left colon cancer (LCC) and rectal cancer (ReC) within mucinous and non-mucinous adenocarcinoma, both with significant differences (both P<0.0001).

There were significant interactions between location and tumor stage, between location and histology, also between location and age on the survival, hence we stratified the results by tumor stage, histology and age. The survival differences between tumor location groups differed according to disease stage (Fig 3). Among stages I and II disease, RCC patients had better DSS than those with ReC; among stage II disease, RCC patients had better DSS than those with LCC. However, among stages III and IV disease, DSS was worse in RCC patients than those with either LCC or ReC, without significant difference between LCC and ReC groups. The survival differences among tumor location groups also differed by histologic type (Fig 4). In patients with non-mucinous tumors, those with LCC or ReC had better DSS than RCC patients; however, in patients with mucinous tumors, those with LCC or ReC had worse DSS than RCC patients (Table 2). DSSs were similar between LCC and ReC groups regardless of histologic type. Finally, the survival differences between tumor location groups varied by age (Table 2). In patients 70 years or younger, RCC patients had worse DSS than those with LCC or ReC. However, in patients older than 70 years, RCC patients had better DSS than those with LCC or ReC. DSSs were similar between LCC and ReC regardless of age group.

## Discussion

In the current study, RCC differed from both LCC and ReC in the distribution of baseline characteristics and in DSS both overall and stratified by disease stage, patient age, and tumor histologic type. Moreover, few significant differences in both the distribution of baseline characteristics and DSS were found between LCC and ReC. Therefore, it seems reasonable to incorporate LCC and ReC into one entity, namely, distal CRC, which differs from RCC, namely, proximal colon cancer.

As supplement of these site-specific differences in clinical features, previous studies found that RCC was prone to lymph node and peritoneal metastases, while LCC and ReC were prone to liver and lung metastases[8, 11, 14]. In other studies, risk factors including black race and a history of cancer or diabetes were closely related to RCC, while factors including white race, smoking, and high alcohol intake were closely associated with both LCC and ReC[15–19]. Recent studies indicated that LCC had higher concentrations of human DNA in the stools than RCC[20], while RCC showed significantly higher mRNA expression levels of glycolysis genes than LCC[21]. Differences were also observed between proximal and distal colon cancers in common alterations of mucosal gene-expression profiles in CRC-associated microbiota [22]. Therefore, sufficient clinical evidence, covering epidemiologic features, clinicopathologic features, survival, and genetic variations, supports the proximal and distal division of CRC.

Some previous studies have indicated that RCC patients have worse survival than those with distal CRC[23, 24]. In agreement, our study found worse DSS in RCC patients than in patients with distal CRC. Furthermore, DSS was significantly worse in patients with RCC than in patients with distal CRC in the subgroups of patients who had stage III or IV disease, were 70 years or younger, or had non-mucinous adenocarcinoma. The predictive value of primary tumor location in survival among patients with metastatic CRC was confirmed in several latest studies[25–28]. Chen et al revealed that right-sided tumor site was an independent predictor of shorter PFS (HR = 1.32, P = 0.0072) and OS (HR = 1.45, P = 0.0003) among 969 patients with KRAS wild-type CRC receiving Cetuximab therapy treatment[26]. In agreement with it, Moretto et al’s study indicated that patients with right-sided RAS and BRAF wild-type metastatic CRC derived no benefit from single Cetuximab therapy (p<0.0001) [27]. Another study determined the impact of primary tumor site on Bevacizumab efficacy in 638 patients with metastatic CRC and indicated median overall survivals of 18.2, 23.6 and 26.2 months in RCC, LCC and ReC patients, respectively (P = 0.0004)[28]. These studies suggested a potential role for primary tumor location in driving treatment choices.

However, RCC patients were observed to have better outcomes than those with LCC and ReC in some specific subgroups. Firstly, RCC had better outcome at stage II in comparison with LCC and better outcomes at stages I and II in comparison with ReC. In other words, RCC was associated with a relatively favorable outcome in early-stage disease, but opposite in regional and metastatic disease. The similar results have been found in previous studies[14, 29]. Here, tumor heterogeneity was considered as the main potential reason. It’s acknowledged that tumor cells with specific genotype have clonal advantages intra a single tumor, and the advantageous genotype may vary responding to environment and treatments. Tumor phenotypes may vary with process of tumor infiltration and metastasis[30, 31]. For instance, mismatch repair deficiency (MMR) was associated with early-stage, non-metastatic CRC[32]. Also, expression of MHC-I has been found to decrease with tumor growth and invasion in CRC, with corresponding decreases in survival[33]. These tumor growth–associated variations could contribute to the stage-dependent differences in outcome we saw between RCC and distal CRC. It’s supposed that malignancy degree may increase with increasing disease stage for RCC. Consequently, it is important to make an early diagnosis in CRC, especially RCC, not only to secure an opportunity for curative resection but also to strive for a relatively low-degree malignancy. In addition, a multivariate analysis of 543 patients with T1 or T2 CRCs indicated that tumor location was an independent prognostic factor, however MMR defect, p53 expression and microsatellite instability were not[34]. Indeed, we observed inferior outcomes for ReC compared with LCC or RCC among stages I and II disease. Tumor location was predictive of prognosis in early-stage CRCs, though the role of underlying genetic variations could not be excluded. This phenomenon may be associated with the special anatomic structure of and treatment methods for ReC. Some rectum segment is directly adjacent to surrounding pelvic organs without coverage by serosa, and rectal tumors within submucosa are removed by transanal endoscopic microsurgery in association of a good life quality. T1 ReC patients had a higher recurrence rate after transanal endoscopic microsurgery than after radical surgery (13.2% vs. 2.7%)[35, 36]. These results reminded us to find the best balance between life quality and survival benefit by choosing a suitable therapeutic intensity for patients with early-stage ReC.

Secondly, RCC patients had better DSS than those with distal CRC among patients with mucinous CRC. The special interaction between tumor location and histologic type among CRC was another important finding in this study. From an opposite perspective, one study found that compared to non-mucinous tumors, mucinous tumors were associated with better outcomes among RCC (*P*< 0.001), and with similar outcomes among LCC[37], while another study found similar survivals between two histology types among ReC[38],in agreement with the current study.

Thirdly, RCC patients had better survival than those with distal CRC among patients older than 70 years. In older patients, CRCs result mainly from external causes including long-time exposure to carcinogenic agents, decreased immunity, and communication of genetic variations. Carcinogenetic agents in the local environment accumulate more in the distal colorectum than in the proximal colon. Tumor microenvironments were also different according to tumor location, with more peritumor lymphocytes and more tumor-infiltrating lymphocytes in proximal tumors than in distal ones[39]. All these factors may contribute to the superior outcomes in older patients with RCC. In fact, among patients older than 50 years, outcomes improved with age for RCC compared with distal CRC. In summary, RCC patients had better outcomes than those with distal CRC in several subgroups including stage II disease, patients aged ˃70 years and mucinous adenocarcinoma. Tumor heterogeneity and different carcinogenesis may make contributions.

In two studies of mCRC, patients with right-sided cancer had worse outcomes than those with left-sided cancer[23, 24]. A study of CRC patients who received surgery[13] found that compared to patients with left-sided cancer, those with right-sided cancer had similar outcomes when they had non-metastatic disease. Another two studies based on the SEER database, focused only on colon cancer, compared RCC and LCC at each stage[14, 29]. In agreement with the present study, survival differences according to tumor location varied by disease stage.

Compared with these previous studies, this study had the strength of better representing the entity of CRC, including stages I to IV and both colon and rectal cancer. Patients enrolled in this study were diagnosed in relatively recent years, from 2002 to 2012, and so their outcomes are comparable with those of CRC patients today. On the basis of detailed comparisons between the three tumor location groups, this study indicated that the proximal and distal division is the most reasonable one.

Our study had some limitations. Because it was a retrospective investigation using a population-based database, we were not able to account for either genetic or lifestyle factors linked with CRC, including testing for MMR or P53 status, or other driver-gene mutations. Data was lacked on subsequent systemic treatment. This prevented us from evaluating these factors as potential confounders or effect modifiers in the observed relationships.

In conclusion, LCC and ReC share characteristics but differ from RCC with regard to baseline characteristics and DSS. Tumor location played a role in predicting survival; compared with patients with LCC or ReC, RCC patients had worse survival especially in subgroups including stage III or IV disease, age ≤70 years and non-mucinous adenocarcinoma. Accordingly, it seems reasonable to divide CRC into right-sided (i.e., proximal) and left-sided (i.e., distal) cancers.

## Supporting Information

S1 Table Baseline characteristics by primary tumor location in random sample dataset. (DOCX)

S2 Table Cox proportional hazards analysis of influence on disease-specific survival. (DOCX)

S3 Table Cox proportional hazards analysis of influence on disease-specific survival by stage, age at diagnosis, and histologic type in random sample dataset. (DOCX)
