# Supplementary material for: Characteristics of Differently Located Colorectal Cancers Support Proximal and Distal Classification: A Population-Based Study of 57,847 Patients
Source: PLoS One. 2016 Dec 9;11(12):e0167540. doi: 10.1371/journal.pone.0167540 (PMC5147913; doi:10.1371/journal.pone.0167540)
Supplement: S1 Table — (DOCX) [file pone.0167540.s001.docx]

**S1 Table. Baseline characteristics by primary tumor location in random sample dataset.**

| Characteristic | RCC (%) (n=12591) | LCC (%) (n=10896) | ReC (%) (n=10875) | P |
| --- | --- | --- | --- | --- |
| Age at diagnosis, years |  |  |  | 0.0001^ |
| Median (range) | 75 (17-108) | 69 (18-102) | 68 (20-107) |  |
| ≤50 | 6.2 | 11.7 | 14.0 |  |
| 51-60 | 11.6 | 18.9 | 19.9 |  |
| 61-70 | 19.9 | 22.8 | 22.3 |  |
| 71-80 | 31.0 | 27.9 | 24.7 |  |
| 81+ | 31.2 | 18.7 | 19.1 |  |
| Year of diagnosis |  |  |  | <0.0001 |
| 2000-2004 | 49.0 | 51.3 | 47.9 |  |
| 2005-2008 | 27.0 | 26.3 | 27.8 |  |
| 2009-2012 | 24.0 | 22.4 | 24.3 |  |
| Ethnicity |  |  |  | <0.0001 |
| Non-Hispanic white | 75.3 | 70.5 | 73.8 |  |
| Black | 12.8 | 11.5 | 9.3 |  |
| Hispanic white | 5.1 | 6.0 | 6.3 |  |
| Asian | 6.3 | 11.1 | 9.9 |  |
| Others | 0.5 | 0.9 | 0.7 |  |
| Sex | | | | <0.0001 |
| Female | 55.1 | 47.3 | 41.5 |  |
| Male | 44.9 | 52.7 | 58.5 |  |
| Stage |  |  |  | <0.0001* |
| 0 | 2.6 | 4.6 | 4.7 |  |
| I | 22.1 | 28.6 | 32.3 |  |
| II | 31.7 | 24.7 | 21.4 |  |
| III | 26.7 | 25.0 | 25.4 |  |
| IV | 16.8 | 17.5 | 16.1 |  |
| Tumor grade |  |  |  | <0.0001* |
| I | **9.4** | 12.2 | 10.1 |  |
| II | 65.6 | 73.6 | 73.6 |  |
| III | 23.2 | 13.6 | 15.4 |  |
| Undifferentiated | 1.8 | 0.6 | 0.9 |  |
| Mucinous histology |  |  |  | <0.0001 |
| No | 84.4 | 82.9 | 92.8 |  |
| Yes | 15.6 | 7.1 | 7.2 |  |
| Primary surgery | | | | <0.0001* |
| Not performed | 8.7 | 9.4 | 20.9 |  |
| Performed | 91.3 | 90.6 | 79.1 |  |

Abbreviations: RCC, right colon cancer; LCC, left colon cancer; ReC, rectal cancer. *Exclude unknown category; ^Kruskal-Wallis equality-of-populations rank test
