# Supplementary material for: Characteristics of Differently Located Colorectal Cancers Support Proximal and Distal Classification: A Population-Based Study of 57,847 Patients
Source: PLoS One. 2016 Dec 9;11(12):e0167540. doi: 10.1371/journal.pone.0167540 (PMC5147913; doi:10.1371/journal.pone.0167540)
Supplement: S2 Table — (DOCX) [file pone.0167540.s002.docx]

**S2 Table. Cox proportional hazards analysis of influence on disease-specific survival.**

| Variable | HR | SE | P | 95% CI | |
| --- | --- | --- | --- | --- | --- |
| Location |  |  |  |  |  |
| RCC | Referent |  |  |  |  |
| LCC | 0.87 | 0.02 | <0.0001 | 0.84 | 0.90 |
| ReC | 0.94 | 0.02 | 0.02 | 0.90 | 0.99 |
| Mucinous histology |  |  |  |  |  |
| No | Referent |  |  |  |  |
| Yes | 1.07 | 0.03 | 0.02 | 1.01 | 1.12 |
| Stage |  |  |  |  |  |
| I | Referent |  |  |  |  |
| II | 2.18 | 0.08 | <0.0001 | 2.03 | 2.35 |
| III | 4.24 | 0.15 | <0.0001 | 3.95 | 4.54 |
| IV | 20.93 | 0.74 | <0.0001 | 19.53 | 22.42 |
| Tumor grade |  |  |  |  |  |
| I | Referent |  |  |  |  |
| II | 1.01 | 0.04 | 0.82 | 0.94 | 1.08 |
| III | 1.45 | 0.05 | <0.0001 | 1.34 | 1.56 |
| Undifferentiated | 1.52 | 0.11 | <0.0001 | 1.31 | 1.76 |
| Race/ethnicity |  |  |  |  |  |
| Non-Hispanic white | Referent |  |  |  |  |
| Black | 1.19 | 0.03 | <0.0001 | 1.13 | 1.25 |
| Hispanic white | 0.88 | 0.03 | 0.001 | 0.81 | 0.95 |
| Asian | 0.91 | 0.03 | 0.005 | 0.86 | 0.97 |
| Others | 0.53 | 0.08 | <0.0001 | 0.39 | 0.72 |

Abbreviations: RCC, right colon cancer; LCC, left colon cancer; ReC, rectal cancer; HR: hazard ratio; SE: standard error; CI: confidence interval
