# Supplementary material for: Characteristics of Differently Located Colorectal Cancers Support Proximal and Distal Classification: A Population-Based Study of 57,847 Patients
Source: PLoS One. 2016 Dec 9;11(12):e0167540. doi: 10.1371/journal.pone.0167540 (PMC5147913; doi:10.1371/journal.pone.0167540)
Supplement: S3 Table — (DOCX) [file pone.0167540.s003.docx]

**Table S3**. **Cox proportional hazards analysis of influence on disease-specific survival by stage, age at diagnosis, and histologic type in random sample dataset.**

|  | | LCC vs. RCC | | | | ReC vs. RCC | | | | ReC vs.LCC | | |
| --- | --- | --- | --- | --- | --- | --- | --- | --- | --- | --- | --- | --- |
|  | | HR (95%CI) | | P | | HR (95%CI) | | P | | HR (95%CI) | | P |
| Overall | | 0.85 (0.81-0.88) | | <0.0001 | | 0.87 (0.83-0.92) | | <0.0001 | | 1.04 (0.98-1.08) | | 0.2 |
| By stage | | | | | | | | | | | | |
| I | 0.68 (0.56-0.82) | | <0.0001 | | 1.25 (1.06-1.47) | | 0.009 | | 1.09 (0.96-1.24) | | 0.2 | |
| II | 1.22 (1.09-1.37) | | 0.001 | | 1.32 (1.17-1.50) | | <0.0001 | | 1.09 (0.96-1.24) | | 0.2 | |
| III | 0.74 (0.68-0.82) | | <0.0001 | | 0.78 (0.71-0.86) | | <0.0001 | | 1.06 (0.96-1.17) | | 0.3 | |
| IV | 0.77 (0.71-0.83) | | <0.0001 | | 0.71 (0.66-0.77) | | <0.0001 | | 0.93 (0.86-1.01) | | 0.1 | |
| By histologic type | | | | | | | | | | | | |
| Mucinous | 1.22 (1.06-1.40) | | 0.004 | | 1.20 (1.04-1.38) | | 0.009 | | 0.99 (0.84-1.16) | | 0.9 | |
| Non-mucinous | 0.83 (0.78-0.87) | | <0.0001 | | 0.85 (0.81-0.90) | | <0.0001 | | 1.04 (0.99-1.10) | | 0.2 | |
| By age at diagnosis (years) | | | | | | | | | | | | |
| ≤50 | 0.90(0.76-1.07) | | 0.2 | | 0.89 (0.76-1.07) | | 0.2 | | 0.99 (0.86-1.14) | | 0.9 | |
| 51-60 | 0.71 (0.62-0.80) | | <0.0001 | | 0.71 (0.63-0.81) | | <0.0001 | | 1.02 (0.90-1.15) | | 0.8 | |
| 61-70 | 0.78 (0.70-0.87) | | <0.0001 | | 0.82 (0.74-0.92) | | 0.001 | | 1.06 (0.95-1.19) | | 0.3 | |
| 71-80 | 0.95 (0.87-1.04) | | 0.2 | | 1.02 (0.93-1.12) | | 0.7 | | 1.08 (0.98-1.19) | | 0.1 | |
| 81+ | 1.14 (1.04-1.24) | | 0.006 | | 1.14 (1.04-1.24) | | 0.004 | | 1.01 (0.91-1.12) | | 0.8 | |

The multivariate analysis was adjusted for race, tumor grade, and sex.

Abbreviations: RCC, right colon cancer; LCC, left colon cancer; ReC, rectal cancer; HR, hazard ratio; CI, confidence interval.
